# The Knockout of Protocadherin Gamma C3 (PCDHGC3) in Breast Cancer and Melanoma Cell Lines Leads to Increased Adhesion of Knockout Cells to Brain Microvascular Endothelial Cells

**DOI:** 10.3390/neurosci7020047

**Published:** 2026-04-18

**Authors:** Paul Glogau, Junqiao Mi, Patrick Meybohm, Malgorzata Burek

**Affiliations:** 1 Department of Anaesthesiology, Intensive Care, Emergency and Pain Medicine, University Hospital Würzburg, 97080 Würzburg, Germany; 2 Graduate School of Life Sciences, Julius-Maximilians-Universität Würzburg, 97074 Würzburg, Germany

**Keywords:** brain microvascular endothelial cells, metastases, adhesion, protocadherin gamma C3 (PCDHGC3), breast cancer, melanoma

## Abstract

Brain metastases remain a major problem for cancer patients, impacting their treatment and survival. The pathogenesis of brain metastases is largely unknown. Recent reports indicate that the adhesion molecule protocadherin γ C3 (PCDHGC3) is differentially expressed in various cancer cells and endothelial cells of the blood–brain barrier (BBB), suggesting its involvement in the development of brain metastases. Therefore, we generated a PCDHGC3 knockout (KO) in the triple-negative breast cancer cell line HCC1806 and the malignant melanoma cell line A2058. Control and KO cells were compared using cell proliferation, adhesion and invasion assays, gene expression analyses and matrix metalloproteinase (MMP) activity assays. While the PCDHGC3 KO mutation led to increased proliferation in HCC1806 cells, with no difference observed in A2058, it significantly increased adhesion to in vitro BBB models as well as invasion in both HCC1806 and A2058 KO cell lines. Although changes in mRNA expression of genes involved in metastasis, angiogenesis and cell adhesion were found in PCDHGC3 KO breast cancer and melanoma cells, the number of genes with significantly increased mRNA expression was higher in A2058 KO cells than in HCC1806 KO cells. While the mRNA expression of MMP1 and 2 was increased in A2058 KO cells, no significant changes were found in HCC1806 KO cells. However, increased MMP activity in the cell culture medium was detected in HCC1806 KO cells, while A2058 KO cells showed lower MMP-activity compared to control. These findings provide insights into the role of PCDHGC3 in cancer cell extravasation during metastatic process and identify potential therapeutic targets for further investigation.

## 1. Introduction

The brain, as the central control organ of our body, requires the maintenance of a specific environment and a continuous, sufficient supply of oxygen and other important substances in order to fulfill its function [1]. At the same time, it must be protected from the harmful substances in the bloodstream, and metabolic waste products must be removed. The highly specialized blood–brain barrier (BBB), a complex of endothelial cells, basement membrane, pericytes and astrocyte endfeet is maintaining this balance. The actual barrier function is performed by the brain microvascular endothelial cells (BMECs), which are interconnected by a complex network of cell–cell contacts, particularly tight junctions (TJ) [2].

Breast cancer and malignant melanoma, along with lung cancer, are the most common causes of brain metastases [3]. Cerebral metastases are found in 10–16% of all breast cancer patients in advanced stages; autopsy studies show a rate of up to 30% [4]. In melanoma, brain metastases can be detected in 40–50% of affected patients with high-grade tumors during the course of the disease. In postmortem autopsies, cerebral metastases have been found in up to 80% of cases [5]. Since brain metastases are associated with limited treatment options, a low quality of life and short overall survival, understanding the underlying pathogenesis and evaluating diagnostic and treatment options is crucial [6,7].

The central nervous system (CNS) metastasis requires tumor cells first adhere to the luminal side of brain capillaries and then cross the BBB. Identifying the molecules that promote adhesion to BMECs is a critical focus of research [8,9]. Recent studies have identified EGFR, MMP1, COX2 and VLA-4 as key mediators of this process [10,11,12]. Concurrently, loss of E-cadherin function in tumor cells increases invasiveness and enhances metastatic potential [13]. Protocadherins, with over 80 isoforms and strong expression in the CNS, constitute the largest subgroup of cadherins. Protocadherin γ C3 (PCDHGC3) could potentially act as a tumor suppressor, as it inhibits the β-catenin/Wnt and mTOR signaling pathways [14,15]. Methylation of the PCDHGC3 promoter, leading to silencing of PCDHGC3 expression, has been described in colorectal carcinoma and intestinal neuroendocrine carcinomas [14,16]. We recently investigated the effects of PCDHGC3 deletion in BMECs and found that barrier integrity was compromised and gene expression was changed [17,18]. In glioblastoma cell line, however, deletion of PCDHGC3 resulted in a slower proliferation rate but a significantly accelerated migration rate [19].

To further investigate the role of PCDHGC3 in cancer and the interaction of cancer cells with the BBB, we generated PCDHGC3 knockout cells using breast cancer and melanoma cell lines. We used the triple-negative breast cancer cell line HCC1806 and the melanoma cell line A2058. Two in vitro BBB models, hCMEC/D3 and human brain-like endothelial cells (BLECs) derived from CD34-positive hematopoietic stem cells, were used in adhesion assays [20,21,22]. Both in vitro BBB models are well characterized, express endothelial and BBB marker proteins and have been used in studies on the mechanisms of metastatic extravasation of cancer cells [10,21,23]. Deletion of PCDHGC3 led to altered gene expression and stronger adhesion of cancer cells to BMECs as well as invasion, suggesting an active role for PCDHGC3 in the metastatic extravasation of cancer cells across the BBB.

## 2. Materials and Methods

### 2.1. Cell Culture

Triple-negative breast cancer cell line HCC1806 (CRL-2335, ATCC, Manassas, VA, USA) was cultured in RPMI medium (R7509-500ML, Sigma-Aldrich, St. Louis, MO, USA) containing 10% FCS, L-glutamine and penicillin/streptomycin. Melanoma cell line A2058 (CRL-3601, ATCC, Manassas, VA, USA) was cultured in Minimum Essential Medium Eagle (MEM) (M5650) containing 2% MEM Non-essential-Amino Acid Solution (M7145, Sigma-Aldrich, St. Louis, MO, USA), 10% FCS, L-glutamine and penicillin/streptomycin. CD34+ hematopoietic stem cells were isolated according to established protocols and differentiated into brain-like endothelial cells (BLECs) in co-culture with human brain immortalized pericytes (CL 05008CLTH, CELTHER Polska Ltd., Konstantynow Lodzki, Poland) [20,21,24]. BLECs were cultured in Endothelial Cell Basal Medium (ECM), supplemented with the Microvascular Endothelial Cell Growth Supplement Kit containing 5% FCS (PB-BH-100-9806, PB-SH-100-4099, PELOBiotech, Planegg, Germany). The human immortalized brain microvascular endothelial cell line hCMEC/D3 (SCC066, Merck Millipore, Darmstadt, Germany) was cultured in Endothelial Cell Basal Medium, supplemented with the Microvascular Endothelial Cell Growth Supplement Kit containing 5% FCS (PB-BH-100-9806, PB-SH-100-4099, PELOBiotech, Planegg, Germany).

### 2.2. Generation of PCDHGC3 Knockout Cell Lines

The level of PCDHGC3 protein was investigated in various breast cancer cell lines and the melanoma cell line A2058 (Supplementary Figure S1). HCC1806 and A2058 with high PCDHGC3 protein expression were selected for transfection. The cells were co-transfected with the PCDH2 CRISPR/Cas9 KO Plasmid (h) (sc-406270, Santa Cruz Biotechnology, Dallas, TX, USA) and the PCDH2 HDR Plasmid (h2) (sc-406270-HDR-2, Santa Cruz Biotechnology, Dallas, TX, USA) using Lipofectamine 3000 (L3000001, Thermo Fisher Scientific, Waltham, MA, USA). Control cells were transfected with PCDH2 HDR Plasmid (h2) only. Transfected clones were selected with 3 µg/mL puromycin, and knockout efficiency was verified by Western blot, as described below.

### 2.3. Western Blot

Cells were washed twice with PBS and lysed with RIPA buffer (50 mM Tris pH8.0, 150 mM NaCl, 0.1% SDS, 0.5% sodium deoxycholate, 1% NP40) containing protease inhibitors (cOmplete Ultra Tablets Mini, Roche, Basel, Switzerland). The lysates were homogenized and centrifuged for 10 min at 4 °C and 10,000× *g*. Protein concentration was determined using the Pierce^TM^ BCA Protein Assay Kit (23225, Thermo Fisher Scientific, Waltham, MA, USA). Equal amounts of protein lysates (5 µg) mixed with NuPAGE^TM^ LDS Sample Buffer (4×) were loaded to NuPAGE^TM^ 4–12%, Bis-Tris, 1.0 mm Mini Protein Gel (NP0321BOX, Thermo Fisher Scientific, Waltham, MA, USA). After electrophoresis, the proteins were transferred to a PVDF membrane (Bio-Rad Laboratories) and blocked with 5% (*w*/*v*) non-fat milk in PBS. The membranes were incubated overnight at 4 °C with primary antibodies diluted in PBS with 1% bovine serum albumin (Sigma-Aldrich, St. Louis, MO, USA). The following antibodies were used: monoclonal anti-gamma-protocadherin C3 antibody (1:1000, 75-237, NeuroMab, Davis, CA, USA), monoclonal anti-β-actin antibody (1:20,000, A3854, Sigma-Aldrich, St. Louis, MO, USA) and an HRP-conjugated secondary anti-mouse IgG antibody (1: 3000, 7076, Cell Signaling Technology, Danvers, MA, USA). The images were acquired using an enhanced chemiluminescence solution and the FluorChem FC2 Multi-Imager II (Alpha Innotech, San Leandro, CA, USA).

### 2.4. BrdU Cell Proliferation Assay

To determine the effects of the PCDHGC3 KO mutation on tumor cell proliferation, KO and control cells were analyzed using the BrdU Cell Proliferation Assay (QIA58, Merck Millipore, Darmstadt, Germany) according to the manufacturer’s protocol. Cells were seeded into 96 well plates at a density of 1000 cells per well and cultured for 24 h. BrdU signals were quantified by spectrometric measurement at 450 nm with a reference wavelength of 540 nm using the Infinite M Plex microplate reader (Tecan).

### 2.5. Cell Adhesion Assay

Endothelial cells were seeded at a density of 4 × 10^4^ cells per well onto gelatin-coated 96 well plates. BLECs were cultured for 5 days in pericyte-conditioned medium, with medium change every 24–48 h. hCMEC/D3 cells were cultured for at least 24 h as described above. For the last 24 h before the experiment, the medium was replaced with ECM containing only L-glutamine and 0.5% charcoal stripped FCS. Tumor cells were trypsinized and fluorescently labeled using the Vybrant^TM^ CFDA SE Cell Tracer Kit (V12883, Thermo Fisher Scientific, Waltham, MA, USA) according to the manufacturer’s instructions. The labeled cancer cells were added to the endothelial cell monolayers at a density of 2.5 × 10^3^ cells per well and incubated at 37 °C for 30, 60 and 120 min to allow adhesion. The wells were washed with PBS, and the fluorescence signal was measured at 492 nm and 535 nm using the Infinite M Plex microplate reader (Tecan).

### 2.6. Transwell Invasion Assay

Control and PCDHGC3 KO cells were seeded at a density of 50 × 10^4^/mL cells in 12 well Transwells (pore size 8 µm, 353182, Corning, Inc., Corning, NY, USA) coated with Matrigel (Corning^®^ Matrigel^®^ Growth Factor Reduced Basement Membrane Matrix, 354230, Corning, Inc., Corning, NY, USA; dilution 1:10 in cell culture medium, coating for 1 h at 37 °C) in cell culture medium described under 2.1. After the cells adhered, the medium in the Transwell insert was replaced with serum-free medium, while normal growth medium with 10% FCS was present in the lower compartment. The cells invaded through the extracellular matrix and migrated through the pores of the Transwell membrane. After 48 h, the cells on the top of the Transwell were removed using a cotton swab. Cells that had migrated to the underside of the membrane were fixed with 70% ethanol for 10 min, washed with PBS, and the membranes were then excised and mounted with ProLong^®^ Gold Antifade Reagent with DAPI. To capture all invaded cells, the entire Transwell membrane was photographed with a fluorescence microscope (Microscope Axio Imager.M2, Carl Zeiss AG, Oberkochen, Germany) and analysed using ImageJ software version 1.52a (National Institutes of Health (NIH), Bethesda, MD, USA).

### 2.7. Real-Time PCR

RNA was extracted from control and KO tumor cells using the NucleoSpin^®^ RNA Kit (MN-740955.250, Macherey-Nagel GmbH & Co. KG, Düren, Germany) according to the manufacturer’s instructions. Briefly, cells were seeded at a density of 25 × 10^4^/mL in a 6 well plate and cultured for 24 h. The isolated RNA was stored at −80 °C. After thawing on ice, the concentration was measured using NanoDrop^TM^ (Thermo Fisher Scientific, Waltham, MA, USA). The cDNA synthesis was performed using 1 µg RNA and the High-capacity cDNA Reverse Transcription Kit (Thermo Fisher Scientific, Waltham, MA, USA) according to the manufacturer’s instructions. To analyze the mRNA expression of selected genes involved in metastasis and cell adhesion, we first examined mRNA expression in control and PCDHGC3 KO cells using the TaqMan™ Array, Human Tumor Metastasis, Fast 96-well (4418875, Thermo Fisher Scientific, Waltham, MA, USA) and TaqMan™ Array, Human Extracellular Matrix & Adhesion Molecules, Fast 96-well (4418910, Thermo Fisher Scientific, Waltham, MA, USA) using the TaqMan™ Fast Advanced Master Mix (4444556, Thermo Fisher Scientific, Waltham, MA, USA) according to the manufacturer’s instructions. GADPH served as an endogenous control. Real-time qPCR was performed using the StepOnePlus™ Real-Time PCR System and analyzed with the instrument software (Thermo Fisher Scientific, Waltham, MA, USA). Validation of selected differentially expressed genes was performed using following specific TaqMan^TM^ Gene Expression Assays (Thermo Fisher Scientific, Waltham, MA, USA): CD44 (Hs01075864_m1), ECM1 (Hs00189435_m1), FGFR4 (Hs01106910_g1), FN1 (Hs01549976_m1), ITGA2 (Hs00158127_m1), LAMB1 (Hs01055960_m1), MMP1 (Hs00899658_m1), MMP2 (Hs01548727_m1), MTSS1 (Hs00207341_m1), PCDHGC3 (Hs00260814_s1), S100A4 (Hs00243202_m1), THBS1 (Hs00962908_m1), TIMP1 (Hs01092512_g1), VEGFC (Hs01099203_m1). CANX (Hs01558409_m1) served as an endogenous control. PCR was performed in the QuantStudio^TM^ 7 Flex System and analyzed using QuantStudio™ Real-Time PCR Software v1.7.1 (Thermo Fisher Scientific, Waltham, MA, USA). Differences in gene expression in qPCR were determined using the ∆∆ threshold cycle (Ct) Algorithm [25]. The relative expression differences (RQ) between PCDHGC3 knockout cells and control cells, normalized to a reference gene (GADPH or CANX), were calculated using the formula 2^−∆∆Ct^.

### 2.8. Matrix Metalloproteinase (MMP)-Activity Assay

Control and PCDHGC3 KO cancer cells were seeded in 6 well plates with a density of 25 × 10^4^/mL in a volume of 1.5 mL per well. The appropriate culture medium without additives was used for each cell line. After 24 h incubation, the medium was collected and stored at −80 °C until use. After thawing, MMP activity was measured using the SensoLyte^®^ 520 MMP Substrate Sampler Kit (AS-71170, AnaSpec Inc., Fremont, CA, USA) according to the manufacturer’s instructions. The MMP substrates are fluorescence resonance energy transfer (FRET) peptides that are cleaved by various MMPs in tumor cell culture medium. Each substrate can be cleaved by a different spectrum of MMPs (Table 1). This cleavage leads to the emission of a fluorescence signal, which was detected at 490 nm and 520 nm using the Infinite M Plex microplate reader (Tecan). The microplate reader was calibrated using the reference standards included in the kit. The gain for subsequent measurements was set to 50 based on the highest coefficient of determination (R^2^).

### 2.9. Statistical Analysis

Data analysis and graphical representation were performed using GraphPad Prism 9 (GraphPad Software). Statistical significance was determined using a *t*-test for two variables and a one-way ANOVA for three or more variables. All results with a *p* value < 0.05 were considered significant.

All experiments were repeated at least three times using various control and KO clones of cell lines. Technically demanding assays, such as cell adhesion and invasion, were performed independently by two researchers.

## 3. Results

### 3.1. Generation of PCDHGC3 Knockout in Breast Cancer and Melanoma Cell Lines

**Figure 1:**
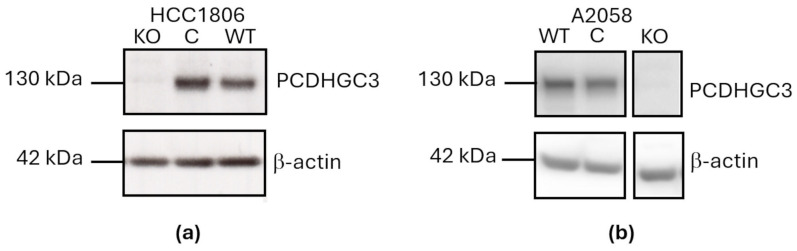
Generation of PCDHGC3 knockout in breast cancer and melanoma cell lines. Western blot analysis of wild type (WT), control (C; transfected with the PCDH2 HDR Plasmid (h2) containing a puromycin resistance gene) and knockout (KO) HCC1806 breast cancer cell line (**a**) and A2058 melanoma cell line (**b**). β-Actin served as an endogenous control.

We investigated the PCDHGC3 protein expression levels in widely used breast cancer cell lines and the melanoma cell line A2058 (Supplementary Figure S1). The breast cancer cell line HCC1806 and the melanoma cell line A2058 showed high PCDHGC3 protein levels and were used to generate PCDHGC3 KO using CRISPR/Cas9 method. To validate the knockout efficiency, a Western blot analysis was performed using an anti-PCDHGC3 antibody, whose specificity had previously been tested on wild type and PCDHGC3 KO BMEC and glioblastoma U343 cells [17,19] (Figure 1). Wild type and control HCC1806 and A2058 cells expressed high levels of PCDHGC3, while no PCDHGC3 protein was detectable in the HCC1806 (Figure 1a) and A2058 cells (Figure 1b) knockout cells. The control and knockout HCC1806 and A2058 cells were used in subsequent experiments.

### 3.2. Deletion of PCDHGC3 in HCC1806 Breast Cancer Cells Leads to Faster Proliferation

**Figure 2:**
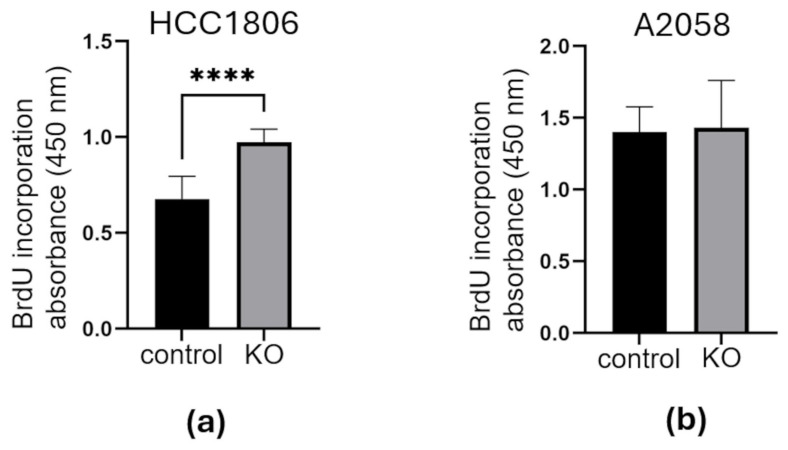
Cell proliferation assay in control and PCDHGC3 knockout cells. Proliferation rate of control and PCDHGC3 knockout (KO) HCC1806 breast cancer cells (**a**) and of control and PCDHGC3 KO A2058 melanoma cells (**b**). **** = *p* < 0.0001, unpaired *t* test.

To characterize the effects of the PCDHGC3 KO mutation on cancer cells, their proliferation rate measured using the BrdU Cell Proliferation Assay (Figure 2). The knockout HCC1806 breast cancer cells proliferated significantly faster than the control cells (1.44 ± 0.07-fold, *p* < 0.0001) (Figure 2a). However, no significant difference in cell proliferation was observed between control and PCDHGC3 KO A2058 melanoma cells (Figure 2b). This suggests that the PCDHGC3-mediated effects vary depending on the cancer type.

### 3.3. PCDHGC3 Knockout Leads to Stronger Adhesion of Tumor Cells to Endothelial Cells and to Stronger Invasion

**Figure 3:**
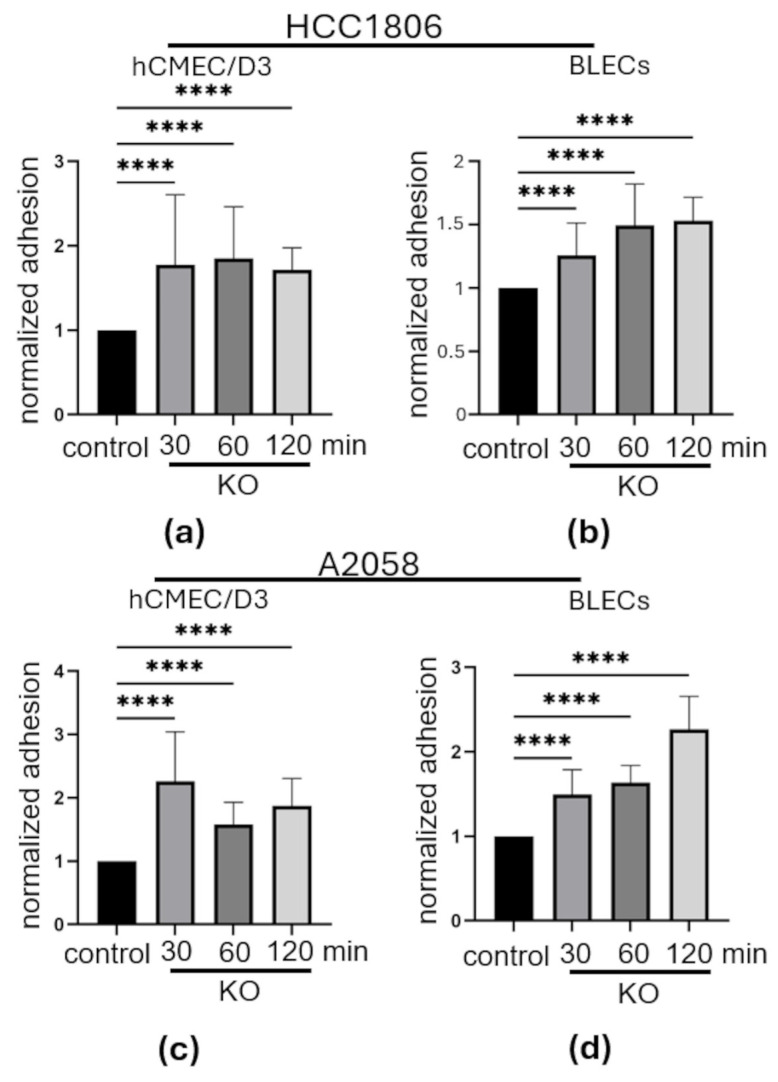
Relative adhesion of PCDHGC3 knockout breast cancer and melanoma cells to human in vitro BBB models. Adhesion measurements of HCC1806 PCDHGC3 knockout (KO) and control cells to hCMEC/D3 (**a**) and BLECs (**b**) after 30, 60, and 120 min. Adhesion measurements of A2058 PCDHGC3 knockout (KO) and control cells to hCMEC/D3 (**c**) and BLECs (**d**) after 30, 60, and 120 min. Control cell adhesion was measured at each time point; however, for clarity, only the measurement after 30 min is shown. Data are presented as mean relative adhesion versus control with standard deviation, **** = *p* ≤ 0.0001, one-way ANOVA test.

We then compared the adhesion of tumor cells to endothelial cells in in vitro BBB models (Figure 3). Two human in vitro BBB models were investigated: The immortalized brain microvascular endothelial cell line hCMEC/D3 and a model based on primary CD34-positive hematopoietic stem cells differentiated into BLECs in co-culture with brain pericytes. In breast cancer cell line HCC1806, the signal of KO cells adhering to hCMEC/D3 cells was on average 1.77 ± 0.13-fold higher after 30 min, 1.85 ± 0.1-fold higher after 60 min, and 1.71 ± 0.05-fold higher after 120 min than that of control cells (*p* < 0.0001 for all measurements). Adhesion to BLECs was 1.27 ± 0.06-fold (*p* = 0.0001) higher in KO cells after 30 min, 1.49 ± 0.07-fold (*p* < 0.0001) higher after 60 min, and 1.55 ± 0.05-fold (*p* < 0.0001) higher after 120 min than in control cells at the respective time points. In summary, while adhesion to BLECs increased with incubation time, it was still lower overall than in the hCMEC/D3 model, where it reached its maximum after 60 min. Overall, the measured fluorescence signal, and thus the adhesion, was significantly stronger in HCC1806 PCDHGC3 KO cells than in control cells (Figure 3a,b).

In A2058, the adhesion of KO cells to hCMEC/D3 cells was 2.19 ± 0.12-fold higher after 30 min, 1.58 ± 0.06-fold higher after 60 min, and 1.87 ± 0.07-fold higher after 120 min than that of control cells (*p* < 0.0001 for all measurements) (Figure 3c). Similarly, the adhesion of A2058 KO cells to BLECs was 1.49 ± 0.05-fold higher after 30 min, 1.63 ± 0.03-fold after 60 min, and 2.27 ± 0.06-fold higher after 120 min (*p* < 0.0001 for all measurements) (Figure 3d). Overall, the fluorescence signal, and thus the number of cancer cells adhering to BLECs, increased continuously with increasing incubation time compared to control cells, with the maximum observed for hCMEC/D3 cells after 30 min. In both in vitro BBB models, the measured relative adhesion of KO cancer cells was significantly increased at all measurement time points compared to control cells (Figure 3). Similar results were achieved with the knockout mouse breast cancer cell line 4T1 and the mouse brain microvascular endothelial cell line (Supplementary Figure S2).

**Figure 4:**
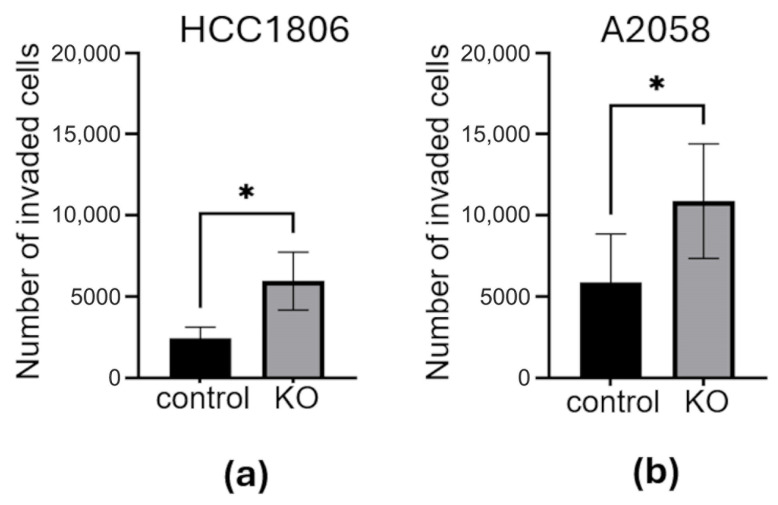
PCDHGC3 KO leads to stronger invasion of PCDHGC3 knockout breast cancer and melanoma cells. HCC1806 PCDHGC3 knockout (KO) and control cells (**a**) and A2058 PCDHGC3 knockout (KO) and control (**b**) invaded for 48 h through Transwells coated with Matrigel. The number of invaded cells is shown. Data are presented as mean cell number with standard deviation, * = *p* ≤ 0.05, unpaired *t*-test.

In addition to adhesion assays, we performed invasion assays in Matrigel-coated Transwell inserts. This assay measures the ability of cells to degrade the extracellular matrix and invade the underside of the membrane, where they are counted. Significantly more PCDHGC3 KO cells invaded the underside of the membrane than the respective control cells in both breast cancer (Figure 4a) and melanoma (Figure 4b).

### 3.4. mRNA Expression Changes Caused by PCDHGC3 Knockout in Tumor Cells

Using the TaqMan™ Array, we identified in HCC1806 breast cancer cells with PCDHGC3 knockout 59 differentially expressed genes of the 171 examined genes with expression changes of at least 20% (Supplementary Table S1). Target genes with a Ct value ≥ 33 or an expression change of less than 20% were excluded, with the exception of VEGFA and MMPs. Among the target genes with altered expression, 13 showed increased expression, while 46 showed decreased expression. Target genes whose expression in KO cells was at least twice as high as in control cells (RQ ≥ 2) were COL1A1, ECM1, and MMP1. Target genes whose relative expression in KO cells at most half as high as in control cells (RQ ≤ 0.5) were B2M, CEACAM1, CNTN1, COL16A1, CXCR4, ICAM1, ITGA4, MMP7, MMP11, MMP16, SNCG, and TNFSF10. When examining the MMPs, it was found that some were overexpressed (MMP-1, -3, -9, -10-, and -12), while the expression of others was reduced (MMP-2, -7, -8, -11, -13, -14, -15, -16).

In PCDHGC3 knockout A2058 melanoma cells, we identified 99 of the 171 target genes examined with expression changes of at least 20% (Supplementary Table S2). The expression changes of MMP3, MMP9, MMP10, MMP12, and MMP13 are shown, although their Ct values were ≥33 either in KO or control cells, indicating low expression in these cells. Among the target genes with altered relative expression, 75 showed increased and 24 showed decreased expression in KO cells. Target genes with a RQ value of at least 2 were ADAMTS1, CD44, COL4A2, COL6A1, COL15A1, FAT1, FGF2, FN1, ITGA2, ITGA4, ITGAM, ITGB5, LAMA3, LAMB1, LAMC1, MMP1, MMP2, MMP15, MMP16, NCAM1, RB1, S100A4, SELL, SPARC, TGFBI, TGFBR2, THBS1, THBS2, TIAM1, TNC, and VCAN. Among these, S100A4 showed 12.7-fold increased expression in KO cells compared with control cells, and VCAN expression was 9.5-fold increased. Targets genes with a RQ value below 0.5 and thus half as high expression in KO cells compared to control cells, were CEACAM1, CNTN1, COL16A1, ECM1, FGFR4, HPSE, IL1B, ITGA1, ITGA7, LAMA1, LAMB3, MET, MTSS1, NR4A3, and PTGS2. Among the MMPs, all except MMP-7 and -13 were overexpressed in KO cells. The expression of MMP-1, -2, -3, -9, -10, -12, -15 and -16 was twice as high in the cells with PCDHGC3 knockout as in the control cells.

**Figure 5:**
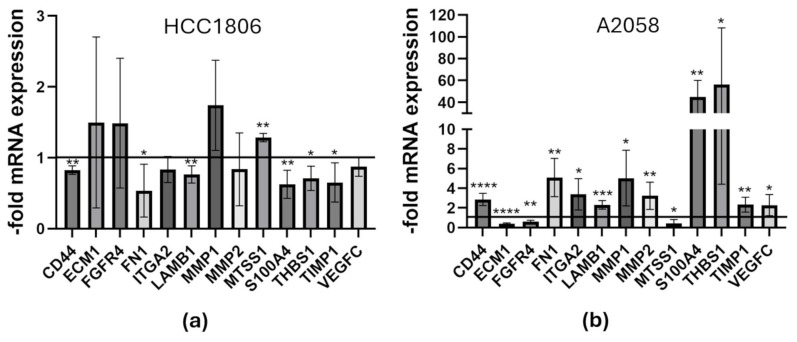
Relative expression of target genes in PCDHGC3 KO breast cancer and melanoma cells. The relative expression (RQ value) of each target in PCDHGC3 KO HCC1806 (**a**) and PCDHGC3 KO A2058 (**b**) cells relative to control cells is shown. A RQ value < 1.0 indicates decreased expression, a RQ value > 1.0 indicates increased expression compared to the control cells. The means with standard deviation are shown as the fold of the control. * = *p* ≤ 0.05, ** = *p* ≤ 0.01, *** = *p* ≤ 0.001, **** = *p* ≤ 0.0001, unpaired *t*-test.

The results obtained with TaqMan™ Array were validated in subsequent qPCR for the selected target genes (Figure 5). In PCDHGC3 KO HCC1806 cells, target genes CD44 (RQ 0.82 ± 0.06), FN1 (RQ 0.54 ± 0.37), LAMB1 (RQ 0.77 ± 0.12), S100A4 (RQ 0.63 ± 0.2), THBS1 (RQ 0.71 ± 0.17) and TIMP1 (RQ 0.65 ± 0.28) showed significantly decreased expression. The KO cells exhibited significantly increased MTSS1 (RQ 1.3 ± 0.06) expression, while target genes ECM1, FGFR4, MMP1 were increased without statistical significance (Figure 5a). In PCDHGC3 KO A2058 melanoma cells, the target genes ECM1, FGFR4, and MTSS1 were expressed at lower levels in PCDHGC3 KO cells compared to control cells. ECM1 (RQ 0.4 ± 0.08) and FGFR4 (RQ 0.59 ± 0.15) were expressed at less than half the level of the control cells. In contrast, the following target genes were overexpressed by the PCDHGC3 KO cells: CD44, FN1, ITGA2, LAMB1, MMP1, MMP2, S100A4, THBS1, TIMP1, and VEGFC. The expression of CD44 (RQ 2.86 ± 0.6), ITGA2 (RQ 3.39 ± 0.08), MMP2 (RQ 3.2 ± 1.38), and VEGFC (RQ 2.28 ± 1.07) was more than twice as high. The KO cells expressed FN1 5.09 ± 1.9-fold, THBS1 56.3 ± 51.86-fold, and S100A4 44.82 ±15.32-fold significantly higher than the control cells (Figure 5b).

### 3.5. MMP Activity in the Tumor Cell Environment Is Influenced by PCDHGC3 Knockout

**Figure 6:**
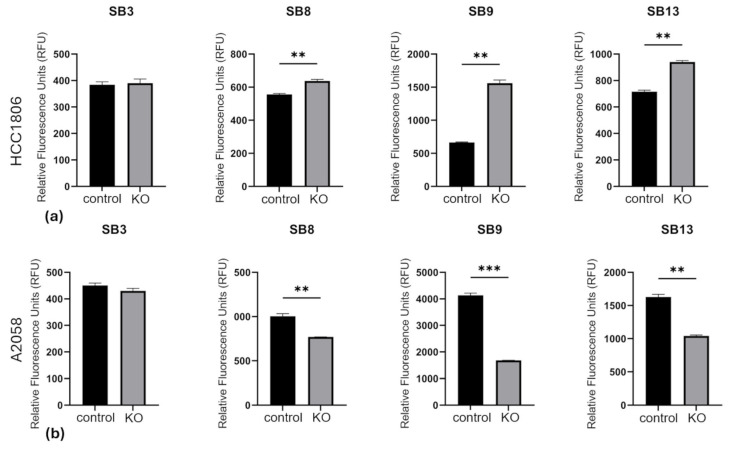
Matrix metalloproteinase (MMP) activity in cell culture medium of PCDHGC3 knockout (KO) breast cancer and melanoma cells. Fluorescence signal of MMP-substrate (SB) cleavage in the cell culture medium of PCDHGC3 KO HCC1806 (**a**) and PCDHGC3 KO A2058 (**b**) cells and control cells is expressed as relative fluorescence units (RFU) ± standard deviation. ** = *p* < 0.01, *** = *p* < 0.001, unpaired *t* test.

Since qPCR results indicated increased expression of selected MMPs, we analyzed protein level by measuring MMP activity in the cell culture medium of the tumor cells (Figure 6). In the culture medium of HCC1806 cells, the MMP activity in PCDHGC3 KO cell culture medium was significantly elevated, as demonstrated with the substrates SB8, SB9 and SB13 (Figure 6a). Incubation with the substrate SB3 did not result in any significant changes compared to the control cells. Considering the substrate specificity (Table 1), it can be assumed that MMP-3 and MMP-7, in particular, are present in elevated concentrations in the cell culture medium. These MMPs cleave SB8 and SB9 (MMP-7) and SB13 (MMP-3), respectively, but not SB3.

In the cell culture medium of the A2058 PCDHGC3 KO cells, a significantly altered fluorescence signal was also observed for the substrates SB8, SB9 and SB13 (Figure 6b). This signal was significantly reduced in every case, indicating lower MMP activity in PCDHGC3 KO cells. No significant changes were observed for SB3. Therefore, it can be assumed that MMP-3 and MMP-7, in particular, are secreted by A2058 cells with PCDHGC3 KO at lower concentrations than by control cells.

## 4. Discussion

Despite many advances in antitumor therapy in general, brain metastases remain a clinical problem with significant morbidity and mortality. After lung cancer, breast cancer and malignant melanoma are the two common cancers that metastasize to the brain [3]. Investigating the pathophysiological processes and the molecules involved is an essential component of efforts to improve the treatability of brain metastases.

The aim of this study was to investigate the adhesion molecule PCDHGC3 and its role in cerebral metastasis. This investigation was conducted using PCDHGC3 knockout cancer cells (HCC1806 breast cancer and A2058 melanoma cells), as well as two established human in vitro BBB models, hCMEC/D3 and BLECs. The use of human models avoids the influence of species-dependent differences in this complex structure. We selected the hCMEC/D3 cell line, established by Weksler et al. in 2005, due to its high stability, good endothelial properties and widespread use in various studies [22,26,27].

The usage of BLECs through the collection and differentiation of CD34^+^ hematopoietic stem cells from umbilical cord blood was first described by Cecchelli et al. and allows for good comparability at comparatively low cost. In addition, the low threshold for obtaining umbilical cord blood enables widespread application. The use of pericytes in culture induces the development of BBB characteristics in the CD34^+^ cells [20,21].

The adhesion of breast cancer cells and malignant melanoma cells was significantly enhanced by PCDHGC3 KO in all measurements and at all time points examined. A similar phenomenon was observed in the cell invasion assay. Thus, these results support the hypothesis that the loss of PCDHGC3 promotes metastasis to the CNS. This is consistent with the assumption that PCDHGC3, along with other PCDHs, acts as a tumor suppressor, but is the only γ-PCDH to do so [15]. To date, no data exist on the role of PCDHGC3 in breast cancer or malignant melanoma. Inactivation of the common gene locus of clustered protocadherins, and thus also of PCDHGC3, on chromosome 5 by hypermethylation is involved in the development of breast cancer, among other mechanisms [28]. In paragangliomas, pheochromocytomas, colon carcinomas, and intestinal neuroendocrine carcinomas, silencing of the PCDHGC3 gene by hypermethylation is significantly involved in tumorigenesis and metastasis [14,16,29]. Increased migration of cerebral endothelial cells following PCDHGC3 knockout is observed, as are increased paracellular permeability and decreased transendothelial electrical resistance, highlighting the potential role of PCDHGC3 in cerebral metastasis [17,18,30]. Breast cancer cell proliferation was significantly higher in cells with a PCDHGC3 KO, consistent with findings in other cell types and cancer cell lines [14,18,29]. Why the PCDHGC3 KO did not alter the proliferation behavior of the A2058 melanoma cell line remains speculative, as no comparative studies are currently available. One possible explanation could be the use of excessively high cell concentrations in the assay. However, assays with varying cell densities showed no differences between KO and control A2058 cells. Overall, A2058 cells grew faster than HCC1806 cells.

At the transcriptional level, the loss of PCDHGC3 led to numerous alterations. Not all of these changes correlate with increased adhesion and metastasis, according to current knowledge. In breast cancer cells, expression of ECM1 and FGFR4 was upregulated. Overexpression of ECM1 in breast cancer is associated with increased metastasis and reduced overall survival and promotes tumor cell migration and invasion by increasing angiogenesis [31,32]. FGFR4 expression is elevated in breast cancer, particularly metastatic breast cancer, and regulates cancer cell survival. As a tyrosine kinase, FGFR4 is also of interest as a potential therapeutic target [33,34].

Overexpression of FN1 reduces metastasis in vivo, which is why the decreased expression of FN1 in PCDHGC3 KO cells could correlate with the promotion of cerebral metastasis [35]. Several genes promoting tumor growth and metastasis were overexpressed by KO cells in malignant melanoma cells; some of these are discussed here. Overexpression of S100A4 promotes the invasiveness and metastasis of malignant melanomas and also leads to endothelial dysfunction of the BBB [36]. VEGFC is involved in lymphatic metastasis and reduces patient survival. Functionally, it promotes tumor cell adhesion by activating TGFβ [37,38,39].

MMPs play diverse roles in tumorigenesis and metastasis. For example, they degrade the extracellular matrix in the tumor microenvironment, thereby promoting invasiveness, promoting angiogenesis, or inhibiting apoptosis [40,41]. MMP-2 and MMP-9 also appear to play an important role in brain metastasis, presumably through the degradation of TJ proteins such as ZO-1, claudin-5, and occludin [42,43,44,45]. In breast cancer cells, the expression of MMP-2 was reduced, while the expression of MMP-1 was increased by PCDHGC3 KO, however without statistical significance. At the protein level, the activity of MMPs, especially MMP-3 and -7, was increased in breast cancer cell lines, while it was decreased in the microenvironment of melanoma PCDHGC3 KO cells. MMP-3 promotes cerebral metastasis of breast cancer [43], while MMP-7 increases the invasiveness of tumor cells. Conversely, MMP-7 reduces cell–cell adhesion [46]; however, a mixture of different MMPs in cell culture medium can influence cell behavior differentially.

In malignant melanoma cells, although the mRNA expression of MMP-1, -2 was significantly increased, the MMP activity in the cellular environment was reduced. MMP-3 is involved in cell invasion and metastasis in malignant melanomas. An important function is the activation of pro-MMP-1 and -13 [47,48,49,50]. MMP-7 expression was significantly reduced, but its role in the development and metastasis of malignant melanoma remains unclear, as published studies show both reduced and increased expression [51,52,53].

In summary, adhesion to brain microvascular endothelial cells and invasion through the extracellular matrix are increased in PCDHGC3 KO cells in breast cancer and melanoma cell lines. However, the PCDHGC3 KO breast cancer cell line HCC1806 showed increased proliferation, while no changes were observed in A2058 PCDHGC3 KO. Measurement of MMP activity revealed increased activity in HCC1806 KO cells but decreased activity in A2058 KO cells. This was also observed in non-overlapping mRNA expression changes of selected genes involved in the metastasis process. According to The Human Protein Atlas, breast cancer cells exhibit low PCDHGC3 expression, while melanoma cells show significantly higher PCDHGC3 expression. These differences in basal PCDHGC3 expression levels could explain the varying effects of the PCDHGC3 KO in these two cell lines. However, more detailed studies with different breast cancer and melanoma cell lines are needed.

A potential limitation of our study lies in the lack of in vivo data on the effect of PCDHGC3 knockout on breast cancer and malignant melanoma metastasis. This restricts the interpretation of our in vitro data. Although our data and those of other groups suggest a likely role for PCDHGC3 in tumorigenesis and metastasis, further research is essential before these findings can be applied clinically.

## Figures and Tables

**Table 1:** Matrix Metalloproteinase substrates of the SensoLyte 520 MMP Substrate Sampler Kit Fluorometric, AnaSpec Inc.

Substrate	Cleaving Enzymes
SB3	MMP-1/2/8/9/12/13
SB8	MMP-7/12/13
SB9	MMP-1/2/7/8/12/13
SB13	MMP-3/12

MMP Matrix Metalloproteinase

## Data Availability

Data are contained within the article and Supplementary Material.

## Supplementary Materials

The following supporting information can be downloaded at: https://www.mdpi.com/article/10.3390/neurosci7020047/s1, Figure S1: Protein expression levels of Protocadherin gamma C3 (PCDHGC3) in cancer cell lines. Breast cancer cell lines (HCC1806, HCC1937, MCF-7, T47-D, MDA-MB 435, ZR 75-1) and a melanoma cell line (A2058) were analyzed in Western blot with anti-PCDHGC3 antibody. β−actin or an unspecific band served as a loading control; Figure S2: Relative adhesion of mouse breast cancer cell line 4T1 to mouse brain microvascular endothelial cells. PcdhgC3 knockout was generated in mouse breast cancer cell line 4T1 and validated by Western blot analysis of control (C) and knockout (KO) 4T1 breast cancer cells. β-actin served as an endogenous control (a). Relative adhesion of control and KO 4T1 cells to mouse brain microvascular endothelial cells after 120 min (b). **** = *p* ≤ 0.0001, unpaired *t* test; Table S1: Differentially expressed genes in HCC1806 breast cancer cells with PCDHGC3 knockout; Table S2: Differentially expressed genes in A2058 melanoma cells with PCDHGC3 knockout.

## Abbreviations

The following abbreviations are used in this manuscript:

BBB	blood–brain barrier
BMEC	brain microvascular endothelial cells
BLEC	brain-like endothelial cells
CNS	central nervous system
FCS	fetal calf serum
MMP	matrix metalloproteinases
PCDHGC3	protocadherin γ C3
PCR	polymerase chain reaction
TJ	tight junction

## References

[1] Safar P., Behringer W., Böttiger B.W., Sterz F. (2002). Cerebral resuscitation potentials for cardiac arrest. Crit. Care Med..

[2] Dithmer S., Blasig I.E., Fraser P.A., Qin Z., Haseloff R.F. (2024). The basic requirement of tight junction proteins in blood-brain barrier function and their role in pathologies. Int. J. Mol. Sci..

[3] Sacks P., Rahman M. (2020). Epidemiology of brain metastases. Neurosurg. Clin..

[4] Lin N.U., Bellon J.R., Winer E.P. (2004). CNS metastases in breast cancer. J. Clin. Oncol..

[5] Qian M., Ma M.W., Fleming N.H., Lackaye D.J., Hernando E., Osman I., Shao Y. (2013). Clinicopathological characteristics at primary melanoma diagnosis as risk factors for brain metastasis. Melanoma Res..

[6] Fabi A., Felici A., Metro G., Mirri A., Bria E., Telera S., Moscetti L., Russillo M., Lanzetta G., Mansueto G. (2011). Brain metastases from solid tumors: Disease outcome according to type of treatment and therapeutic resources of the treating center. J. Exp. Clin. Cancer Res..

[7] Lowery F.J., Yu D. (2017). Brain metastasis: Unique challenges and open opportunities. Biochim. Biophys. Acta (BBA)-Rev. Cancer.

[8] Fares J., Kanojia D., Rashidi A., Ulasov I., Lesniak M.S. (2020). Genes that mediate metastasis across the blood–brain barrier. Trends Cancer.

[9] Reymond N., d’Água B.B., Ridley A.J. (2013). Crossing the endothelial barrier during metastasis. Nat. Rev. Cancer.

[10] Galloni C., Egnuni T., Zahed Mohajerani S., Ye J., Mittnacht S., Speirs V., Lorger M., Mavria G. (2024). Brain endothelial cells promote breast cancer cell extravasation to the brain via EGFR-DOCK4-RAC1 signalling. Commun. Biol..

[11] García-Martín A.B., Zwicky P., Gruber T., Matti C., Moalli F., Stein J.V., Francisco D., Enzmann G., Levesque M.P., Hewer E. (2019). VLA-4 mediated adhesion of melanoma cells on the blood–brain barrier is the critical cue for melanoma cell intercalation and barrier disruption. J. Cereb. Blood Flow Metab..

[12] Wu K., Fukuda K., Xing F., Zhang Y., Sharma S., Liu Y., Chan M.D., Zhou X., Qasem S.A., Pochampally R. (2015). Roles of the cyclooxygenase 2 matrix metalloproteinase 1 pathway in brain metastasis of breast cancer. J. Biol. Chem..

[13] Zeljko M., Pecina-Slaus N., Martic T.N., Kusec V., Beros V., Tomas D. (2011). Molecular alterations of E-cadherin and beta-catenin in brain metastases. Front. Biosci. (Elite Ed.).

[14] Dallosso A., Øster B., Greenhough A., Thorsen K., Curry T., Owen C., Hancock A., Szemes M., Paraskeva C., Frank M. (2012). Long-range epigenetic silencing of chromosome 5q31 protocadherins is involved in early and late stages of colorectal tumorigenesis through modulation of oncogenic pathways. Oncogene.

[15] Mah K.M., Houston D.W., Weiner J.A. (2016). The γ-Protocadherin-C3 isoform inhibits canonical Wnt signalling by binding to and stabilizing Axin1 at the membrane. Sci. Rep..

[16] Cubiella T., Celada L., San-Juan-Guardado J., Rodríguez-Aguilar R., Suárez-Priede Á., Poch M., Dominguez F., Fernández-Vega I., Montero-Pavón P., Fraga M.F. (2024). PCDHGC3 hypermethylation as a potential biomarker of intestinal neuroendocrine carcinomas. J. Pathol..

[17] Dilling C., Roewer N., Förster C.Y., Burek M. (2017). Multiple protocadherins are expressed in brain microvascular endothelial cells and might play a role in tight junction protein regulation. J. Cereb. Blood Flow Metab..

[18] Gabbert L., Dilling C., Meybohm P., Burek M. (2020). Deletion of protocadherin gamma C3 induces phenotypic and functional changes in brain microvascular endothelial cells in vitro. Front. Pharmacol..

[19] Feldheim J., Wend D., Lauer M.J., Monoranu C.M., Glas M., Kleinschnitz C., Ernestus R.-I., Braunger B.M., Meybohm P., Hagemann C. (2022). Protocadherin Gamma C3 (PCDHGC3) is strongly expressed in glioblastoma and its high expression is associated with longer progression-free survival of patients. Int. J. Mol. Sci..

[20] Cecchelli R., Aday S., Sevin E., Almeida C., Culot M., Dehouck L., Coisne C., Engelhardt B., Dehouck M.-P., Ferreira L. (2014). A stable and reproducible human blood-brain barrier model derived from hematopoietic stem cells. PLoS ONE.

[21] Curtaz C.J., Schmitt C., Herbert S.-L., Feldheim J., Schlegel N., Gosselet F., Hagemann C., Roewer N., Meybohm P., Wöckel A. (2020). Serum-derived factors of breast cancer patients with brain metastases alter permeability of a human blood–brain barrier model. Fluids Barriers CNS.

[22] Weksler B., Subileau E., Perriere N., Charneau P., Holloway K., Leveque M., Tricoire-Leignel H., Nicotra A., Bourdoulous S., Turowski P. (2005). Blood-brain barrier-specific properties of a human adult brain endothelial cell line. FASEB J..

[23] Teles R.H.G., Villarinho N.J., Yamagata A.S., Hiroki C.T., de Oliveira M.C., Terçarioli G.R., Jaeger R.G., Meybohm P., Burek M., Freitas V.M. (2025). Valosin-containing protein (VCP), a component of tumor-derived extracellular vesicles, impairs the barrier integrity of brain microvascular endothelial cells. BBA Adv..

[24] Lyck R., Lécuyer M.-A., Abadier M., Wyss C.B., Matti C., Rosito M., Enzmann G., Zeis T., Michel L., García Martín A.B. (2017). ALCAM (CD166) is involved in extravasation of monocytes rather than T cells across the blood–brain barrier. J. Cereb. Blood Flow Metab..

[25] Livak K.J., Schmittgen T.D. (2001). Analysis of relative gene expression data using real-time quantitative PCR and the 2^−ΔΔCT^ method. Methods.

[26] Helms H.C., Abbott N.J., Burek M., Cecchelli R., Couraud P.-O., Deli M.A., Förster C., Galla H.J., Romero I.A., Shusta E.V. (2016). In vitro models of the blood–brain barrier: An overview of commonly used brain endothelial cell culture models and guidelines for their use. J. Cereb. Blood Flow Metab..

[27] Weksler B., Romero I.A., Couraud P.-O. (2013). The hCMEC/D3 cell line as a model of the human blood brain barrier. Fluids Barriers CNS.

[28] Novak P., Jensen T., Oshiro M.M., Watts G.S., Kim C.J., Futscher B.W. (2008). Agglomerative epigenetic aberrations are a common event in human breast cancer. Cancer Res..

[29] Bernardo-Castiñeira C., Valdes N., Celada L., Martinez A.S.J., Sáenz-de-Santa-María I., Bayón G.F., Fernández A.F., Sierra M.I., Fraga M.F., Astudillo A. (2019). Epigenetic deregulation of protocadherin PCDHGC3 in pheochromocytomas/paragangliomas associated with SDHB mutations. J. Clin. Endocrinol. Metab..

[30] Kaupp V., Blecharz-Lang K.G., Dilling C., Meybohm P., Burek M. (2023). Protocadherin gamma C3: A new player in regulating vascular barrier function. Neural Regen. Res..

[31] Gómez-Contreras P., Ramiro-Díaz J., Sierra A., Stipp C., Domann F., Weigel R., Lal G. (2017). Extracellular matrix 1 (ECM1) regulates the actin cytoskeletal architecture of aggressive breast cancer cells in part via S100A4 and Rho-family GTPases. Clin. Exp. Metastasis.

[32] Steinhaeuser S.S., Morera E., Budkova Z., Schepsky A., Wang Q., Rolfsson O., Riedel A., Krueger A., Hilmarsdottir B., Maelandsmo G.M. (2020). ECM1 secreted by HER2-overexpressing breast cancer cells promotes formation of a vascular niche accelerating cancer cell migration and invasion. Lab. Investig..

[33] Levine K.M., Ding K., Chen L., Oesterreich S. (2020). FGFR4: A promising therapeutic target for breast cancer and other solid tumors. Pharmacol. Ther..

[34] Tiong K.H., Tan B.S., Choo H.L., Chung F.F.-L., Hii L.-W., Tan S.H., Khor N.T.W., Wong S.F., See S.-J., Tan Y.-F. (2016). Fibroblast growth factor receptor 4 (FGFR4) and fibroblast growth factor 19 (FGF19) autocrine enhance breast cancer cells survival. Oncotarget.

[35] Glasner A., Levi A., Enk J., Isaacson B., Viukov S., Orlanski S., Scope A., Neuman T., Enk C.D., Hanna J.H. (2018). NKp46 receptor-mediated interferon-γ production by natural killer cells increases fibronectin 1 to alter tumor architecture and control metastasis. Immunity.

[36] Herwig N., Belter B., Pietzsch J. (2016). Extracellular S100A4 affects endothelial cell integrity and stimulates transmigration of A375 melanoma cells. Biochem. Biophys. Res. Commun..

[37] Boone B., Blokx W., De Bacquer D., Lambert J., Ruiter D., Brochez L. (2008). The role of VEGF-C staining in predicting regional metastasis in melanoma. Virchows Arch..

[38] Gajanin V., Krivokuća Z., Kostić K., Gajanin R., Sladojević I. (2010). Significance of vascular endothelial growth factor expression in skin melanoma. Vojnosanit. Pregl..

[39] Hlophe Y.N., Joubert A.M. (2022). Vascular endothelial growth factor-C in activating vascular endothelial growth factor receptor-3 and chemokine receptor-4 in melanoma adhesion. J. Cell. Mol. Med..

[40] Egeblad M., Werb Z. (2002). New functions for the matrix metalloproteinases in cancer progression. Nat. Rev. Cancer.

[41] Kapoor C., Vaidya S., Wadhwan V., Kaur G., Pathak A. (2016). Seesaw of matrix metalloproteinases (MMPs). J. Cancer Res. Ther..

[42] Feng S., Cen J., Huang Y., Shen H., Yao L., Wang Y., Chen Z. (2011). Matrix metalloproteinase-2 and-9 secreted by leukemic cells increase the permeability of blood-brain barrier by disrupting tight junction proteins. PLoS ONE.

[43] Mendes O., Kim H.-T., Stoica G. (2005). Expression of MMP2, MMP9 and MMP3 in breast cancer brain metastasis in a rat model. Clin. Exp. Metastasis.

[44] Rosenberg G.A., Yang Y. (2007). Vasogenic edema due to tight junction disruption by matrix metalloproteinases in cerebral ischemia. Neurosurg. Focus.

[45] Wilhelm I., Molnár J., Fazakas C., Haskó J., Krizbai I.A. (2013). Role of the blood-brain barrier in the formation of brain metastases. Int. J. Mol. Sci..

[46] Ii M., Yamamoto H., Adachi Y., Maruyama Y., Shinomura Y. (2006). Role of matrix metalloproteinase-7 (matrilysin) in human cancer invasion, apoptosis, growth, and angiogenesis. Exp. Biol. Med..

[47] Benbow U., Schoenermark M.P., Mitchell T.I., Rutter J.L., Shimokawa K., Nagase H., Brinckerhoff C.E. (1999). A novel host/tumor cell interaction activates matrix metalloproteinase 1 and mediates invasion through type I collagen. J. Biol. Chem..

[48] Nikkola J., Vihinen P., Vlaykova T., Hahka-Kemppinen M., Kähäri V.M., Pyrhönen S. (2002). High expression levels of collagenase-1 and stromelysin-1 correlate with shorter disease-free survival in human metastatic melanoma. Int. J. Cancer.

[49] Pittayapruek P., Meephansan J., Prapapan O., Komine M., Ohtsuki M. (2016). Role of matrix metalloproteinases in photoaging and photocarcinogenesis. Int. J. Mol. Sci..

[50] Shoshan E., Braeuer R.R., Kamiya T., Mobley A.K., Huang L., Vasquez M.E., Velazquez-Torres G., Chakravarti N., Ivan C., Prieto V. (2016). NFAT1 directly regulates IL8 and MMP3 to promote melanoma tumor growth and metastasis. Cancer Res..

[51] Kawasaki K., Kawakami T., Watabe H., Itoh F., Mizoguchi M., Soma Y. (2007). Expression of matrilysin (matrix metalloproteinase-7) in primary cutaneous and metastatic melanoma. Br. J. Dermatol..

[52] Meng N., Li Y., Jiang P., Bu X., Ding J., Wang Y., Zhou X., Yu F., Zhang Y., Zhang J. (2022). A comprehensive pan-cancer analysis of the tumorigenic role of matrix metallopeptidase 7 (MMP7) across human cancers. Front. Oncol..

[53] Moro N., Mauch C., Zigrino P. (2014). Metalloproteinases in melanoma. Eur. J. Cell Biol..

