# Supplementary material for: The Knockout of Protocadherin Gamma C3 (PCDHGC3) in Breast Cancer and Melanoma Cell Lines Leads to Increased Adhesion of Knockout Cells to Brain Microvascular Endothelial Cells
Source: NeuroSci. 2026 Apr 18;7(2):47. doi: 10.3390/neurosci7020047 (PMC13118706; doi:10.3390/neurosci7020047)
Supplement: Supplementary file 1 [file neurosci-07-00047-s001.zip › neurosci-4186819-supplementary.pdf]

Supplementary Materials

The knockout of protocadherin gamma C3 (PCDHGC3) in breast cancer and melanoma cell lines leads to increased adhesion of knockout cells to brain microvascular endothelial cells

Paul Glogau [1], Junqiao Mi [1,2], Patrick Meybohm [1] and Malgorzata Burek [1,*]

1 Department of Anaesthesiology, Intensive Care, Emergency and Pain Medicine, University Hospital Würzburg, 97080 Würzburg, Germany;
2 Graduate School of Life Sciences, Julius-Maximilians-Universität Würzburg, 97074 Würzburg, Germany
* Correspondence: burek_m@ukw.de

Supplementary Materials and Methods

Generation of PCDHGC3 Knockout in Mouse Cell Lines

Mouse breast cancer cell line 4T1 (CRL-2539, ATCC, Manassas, VA, USA) and the mouse brain microvascular endothelial cell line cerebEND [17, 18] were co-transfected with the Pcdh2 CRISPR/Cas9 KO Plasmid (m) (sc-430015, Santa Cruz Biotechnology, Dallas, TX, USA) and the Pcdh2 HDR Plasmid (m2) (sc-430015-HDR-2, Santa Cruz Biotechnology, Dallas, TX, USA) using Lipofectamine 3000 (L3000001, Thermo Fisher Scientific, Waltham, MA, USA). Control cells were transfected with Pcdh2 HDR Plasmid (h2) only. Transfected clones were selected with 3 μg/ml puromycin and knockout efficiency was verified by Western blot, as described in the main manuscript.

Supplementary Figure S1

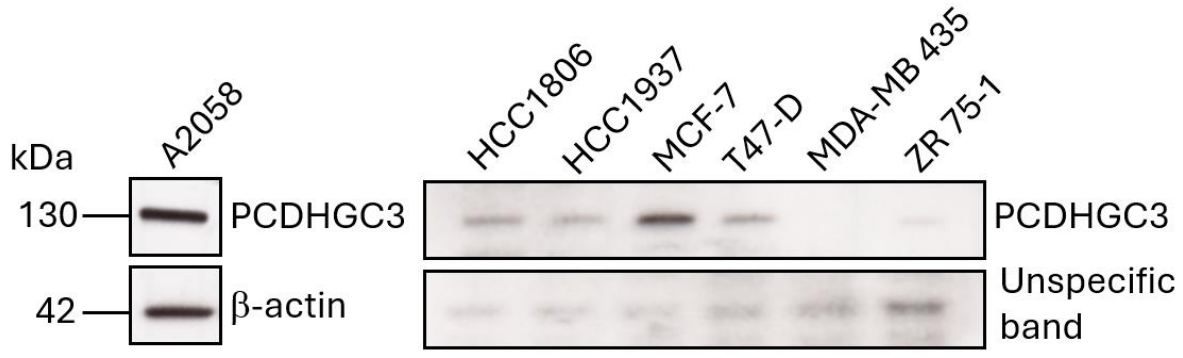

Supplementary Figure S1. Protein expression levels of Protocadherin gamma C3 (PCDHGC3) in cancer cell lines. Breast cancer cell lines (HCC1806, HCC1937, MCF-7, T47-D, MDA-MB 435, ZR 75-1) and a melanoma cell line (A2058) were analyzed in Western blot with anti-PCDHGC3 antibody. β –actin or an unspecific band served as a loading control.

Supplementary Figure S2

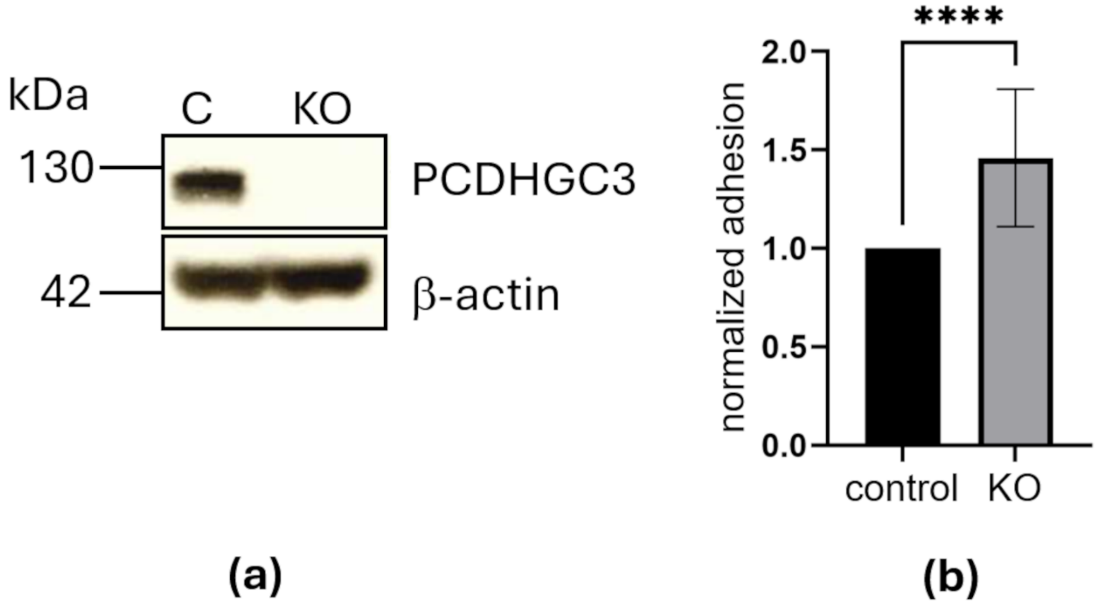

Supplementary Figure S2. Relative adhesion of mouse breast cancer cell line 4T1 to mouse brain microvascular endothelial cells. PcdhgC3 knockout was generated in mouse breast cancer cell line 4T1 and validated by Western blot analysis of control (C) and knockout (KO) 4T1 breast cancer cells. β-actin served as an endogenous control (**a**) Relative adhesion of control and KO 4T1 cells to mouse brain microvascular endothelial cells after 120 minutes (**b**). **** = $p \leq 0.0001$, unpaired t test.

Supplementary Table S1. Differentially expressed genes in HCC1806 breast cancer cells with PCDHGC3 knockout.

Target gene	Name	RQ	Ct KO	Ct control
* 18S	18S rRNA	0.83	8.98	8.73
		0.84	8.98	8.97
	**Mean (RQ)**	**0.83**		
* ACTB	Actin beta	0.89	18.97	18.83
ADAMTS1	ADAM metallopeptidase with thrombospondin type 1 motif 1	0.85	30.98	30.77
ADAMTS13	ADAM metallopeptidase with thrombospondin type 1 motif 13	0.85	30.17	29.95
ADAMTS8	ADAM metallopeptidase with thrombospondin type 1 motif 8	4.85	36.97	39.28
APC	APC regulator of WNT signaling pathway	0.90	25.93	26.03
B2M	beta-2-microglobulin	0.49	19.96	18.95
BRMS1	BRMS1 transcriptional repressor and anoikis regulator	0.72	25.97	25.74
CASP8	caspase 8	0.86	25.92	25.94
CCL7	*C-C motif chemokine ligand 7*	*No amplification*		
CD44	CD44 molecule (Indian blood group)	0.79	21.28	20.95
		0.76	21.10	20.95
	**Mean (RQ)**	**0.77**		
CD82	CD82 molecule	0.54	25.63	24.98
CDH1	cadherin 1	0.87	25.97	25.79
		0.84	24.99	24.98
	**Mean (RQ)**	**0.85**		
CDKN2A	cyclin dependent kinase inhibitor 2A	0.04	45.99	41.58
CEACAM1	CEA cell adhesion molecule 1	0.35	30.96	29.71
CLEC3B	C-type lectin domain family 3 member B	0.84	34.96	34.74
CNTN1	contactin 1	0.50	29.92	28.94
COL11A1	*collagen type XI alpha 1 chain*	*No amplification*		
COL12A1	collagen type XII alpha 1 chain	1.39	23.95	24.46
COL14A1	collagen type XIV alpha 1 chain	4.48	34.79	36.97
COL15A1	*collagen type XV alpha 1 chain*	*No amplification*		*39.99*
COL16A1	collagen type XVI alpha 1 chain	0.21	32.95	30.71
COL1A1	collagen type I alpha 1 chain	2.04	30.93	31.98
COL4A2	collagen type IV alpha 2 chain	0.77	23.96	23.61
COL5A1	collagen type V alpha 1 chain	0.88	25.14	24.98
COL6A1	collagen type VI alpha 1 chain	0.68	26.52	25.99
COL6A2	collagen type VI alpha 2 chain	0.65	25.97	25.37
COL7A1	collagen type VII alpha 1 chain	0.84	24.98	24.75
COL8A1	collagen type VIII alpha 1 chain	0.21	38.99	36.78
CTBP1	C-terminal binding protein 1	0.66	28.99	28.63
CTGF	cellular communication network factor 2	0.92	27.93	27.83

CTNNA1	catenin alpha 1	0.83	23.99	23.74
		0.81	23.01	22.94
	**Mean (RQ)**	**0.82**		
CTNNB1	catenin beta 1	0.91	21.97	21.85
CTNND1	catenin delta 1	0.93	21.96	21.88
CTNND2	catenin delta 1	1.13	38.77	38.98
CTSK	cathepsin K	0.85	28.95	28.96
CXCL12	C-X-C motif chemokine ligand 12	0.57	36.50	35.94
CXCR4	C-X-C motif chemokine receptor 4	0.43	31.96	30.98
DAPK1	death associated protein kinase 1	0.21	35.97	33.98
DCC	*DCC netrin 1 receptor*	*No amplification*		
ECM1	extracellular matrix protein 1	2.81	28.97	30.48
EPCAM	epithelial cell adhesion molecule	0.83	25.99	25.96
EPHB2	EPH receptor B2	0.95	25.96	26.12
ERBB2	erb-b2 receptor tyrosine kinase 2	0.91	25.89	25.99
ETV4	ETS variant transcription factor 4	0.85	26.95	26.95
FAT1	FAT atypical cadherin 1	1.05	22.62	22.93
FGF2	fibroblast growth factor 2	0.84	30.99	30.99
FGFR4	fibroblast growth factor receptor 4	1.21	28.46	28.97
FN1	fibronectin 1	0.75	24.94	24.55
		0.72	24.98	24.75
	**Mean (RQ)**	**0.73**		
FXYD5	FXYD domain containing ion transport regulator 5	0.83	20.94	20.92
* GAPDH	glyceraldehyde-3-phosphate dehydrogenase		19.96	19.98
			19.72	19.96
GNRH1	*gonadotropin releasing hormone 1*	*No amplification*		
* GUSB	glucuronidase beta	1.01	25.93	25.97
		1.09	24.94	25.31
	**Mean (RQ)**	**1.05**		
HAS1	hyaluronan synthase 1	0.46	36.98	35.89
HGF	hepatocyte growth factor	0.31	35.37	33.94
HMBS	hydroxymethylbilane synthase	1.15	26.73	26.96
* HPRT1	hypoxanthine phosphoribosyltransferase 1	1.39	24.96	25.45
		1.29	24.95	25.56
	**Mean (RQ)**	**1.34**		
HPSE	heparanase	1.20	28.97	29.48
HRAS	HRas proto-oncogene. GTPase	1.13	25.55	25.97
HTATIP2	HIV-1 Tat interactive protein 2	0.63	23.38	22.95
ICAM1	intercellular adhesion molecule 1	0.46	27.97	26.87
IGF1	*insulin like growth factor 1*	*No amplification*		*40.95*
IL18	interleukin 18	0.81	24.94	24.89
IL1B	interleukin 1 beta	1.28	26.95	27.55
ITGA1	integrin subunit alpha 1	0.98	28.95	28.95

ITGA2	integrin subunit alpha 2	0.88	24.98	24.83
ITGA3	integrin subunit alpha 3	0.86	29.00	28.80
ITGA4	integrin subunit alpha 4	0.44	30.94	29.77
ITGA5	integrin subunit alpha 5	0.85	25.95	25.74
ITGA6	integrin subunit alpha 6	1.05	20.94	21.03
ITGA7	integrin subunit alpha 7	1.17	31.71	31.96
ITGA8	*integrin subunit alpha 8*	*No amplification*		
ITGAL	integrin subunit alpha L	1.01	33.94	33.97
ITGAM	integrin subunit alpha M	1.59	29.96	30.65
ITGAV	integrin subunit alpha V	0.71	22.94	22.47
ITGB1	integrin subunit beta 1	0.77	24.94	24.59
ITGB2	integrin subunit beta 2	0.21	33.97	31.72
ITGB3	integrin subunit beta 3	0.37	37.36	35.96
		0.91	36.87	36.98
	**Mean (RQ)**	**0.64**		
ITGB4	integrin subunit beta 4	0.84	23.98	23.75
ITGB5	integrin subunit beta 5	0.92	25.97	25.88
KAL1	anosmin 1	0.54	28.83	27.96
KISS1	KiSS-1 metastasis suppressor	0.44	35.89	34.95
KISS1R	KISS1 receptor	0.44	38.94	37.99
KRAS	KRAS proto-oncogene. GTPase	0.89	24.87	24.94
LAMA1	laminin subunit alpha 1	0.24	34.98	32.96
LAMA2	laminin subunit alpha 2	0.90	31.96	31.84
LAMA3	laminin subunit alpha 3	0.64	25.95	25.34
LAMB1	laminin subunit beta 1	0.72	25.39	24.94
		0.70	25.97	25.70
	**Mean (RQ)**	**0.71**		
LAMB3	laminin subunit beta 3	0.78	22.28	21.95
LAMC1	laminin subunit gamma 1	0.79	24.98	24.66
LYPD3	LY6/PLAUR domain containing 3	0.84	24.98	24.97
MCAM	melanoma cell adhesion molecule	1.51	29.97	30.81
MET	MET proto-oncogene, receptor tyrosine kinase	0.73	24.16	23.95
MGAT5	alpha-1,6-mannosylglycoprotein 6-beta-N-acetylglucosaminyltransferase	0.89	25.91	25.98
MMP1	matrix metallopeptidase 1	2.35	25.71	26.97
		2.33	24.49	25.96
	**Mean (RQ)**	**2.34**		
MMP10	matrix metallopeptidase 10	1.14	25.73	25.93
		1.03	25.65	25.93
	**Mean (RQ)**	**1.08**		
MMP11	matrix metallopeptidase 11	0.42	30.97	29.76
MMP12	matrix metallopeptidase 12	1.12	30.79	30.98
MMP13	matrix metallopeptidase 13	0.77	25.98	25.63
MMP14	matrix metallopeptidase 14	0.71	24.97	24.50
		0.67	24.28	23.95

	**Mean (RQ)**	**0.69**		
MMP15	matrix metallopeptidase 15	0.57	29.76	28.98
MMP16	matrix metallopeptidase 16	0.43	32.17	30.96
MMP2	matrix metallopeptidase 2	0.63	25.60	24.97
		0.57	24.53	23.96
	**Mean (RQ)**	**0.60**		
MMP3	matrix metallopeptidase 3	3.03	35.95	37.57
		0.82	35.98	35.94
	**Mean (RQ)**	**1.93**		
MMP7	matrix metallopeptidase 7	0.14	33.78	30.97
		0.17	32.94	30.63
	**Mean (RQ)**	**0.16**		
MMP8	matrix metallopeptidase 8	0.83	35.97	35.74
MMP9	matrix metallopeptidase 9	1.99	32.97	33.98
		1.16	32.49	32.94
	**Mean (RQ)**	**1.57**		
MTA1	metastasis associated 1	0.90	25.89	25.98
MTA2	metastasis associated 1 family member 2	0.81	23.95	23.88
MTSS1	MTSS I-BAR domain containing 1	1.35	25.93	26.61
MYC	MYC proto-oncogene, bHLH transcription factor	1.10	23.97	24.36
NCAM1	neural cell adhesion molecule 1	4.64	31.97	34.21
		10.36	35.36	38.98
	**Mean (RQ)**	**7.50**		
NF2	NF2, moesin-ezrin-radixin like (MERLIN) tumor suppressor	0.95	25.74	25.91
NME1	NME/NM23 nucleoside diphosphate kinase 1	1.34	27.94	28.60
NR4A3	nuclear receptor subfamily 4 group A member 3	0.83	29.99	29.97
PECAM1	platelet and endothelial cell adhesion molecule 1	0.0004	43.99	32.74
		0.0046	43.96	36.44
	**Mean (RQ)**	**0.0025**		
PGK1	phosphoglycerate kinase 1	0.97	23.97	23.95
PNN	pinin, desmosome associated protein	1.08	23.60	23.95
PPIA	peptidylprolyl isomerase A	1.07	19.86	19.98
PSCA	prostate stem cell antigen	0.08	33.28	29.96
PTEN	phosphatase and tensin homolog	0.73	26.96	26.76
PTGS2	prostaglandin-endoperoxide synthase 2	0.83	24.97	24.95
RB1	RB transcriptional corepressor 1	0.88	25.87	25.94
RBL1	RB transcriptional corepressor like 1	1.49	25.12	25.95
RBL2	RB transcriptional corepressor like 2	0.73	25.96	25.76
RET	ret proto-oncogene	0.36	38.99	37.77
RHOC	ras homolog family member C	0.62	28.99	28.54

RPLP0	ribosomal protein lateral stalk subunit P0	0.99	18.94	18.95
S100A4	S100 calcium binding protein A4	0.60	28.95	28.46
SELE	*selectin E*	*No amplification*	*40.98*	
SELL	selectin L	1.20	35.70	35.99
SELP	selectin P	0.54	36.84	35.97
SERPINB5	serpin family B member 5	0.92	22.84	22.97
SERPINE1	serpin family E member 1	0.85	24.98	24.99
SET	SET nuclear proto-oncogene	0.94	24.78	24.93
SGCE	sarcoglycan epsilon	0.88	24.94	24.78
SMAD2	SMAD family member 2	0.87	25.92	25.96
SMAD4	SMAD family member 4	0.94	25.96	26.11
SNCG	synuclein gamma	0.47	24.97	24.13
SPARC	secreted protein acidic and cysteine rich	0.61	35.66	34.97
SPG7	SPG7 matrix AAA peptidase subunit, paraplegin	0.90	24.97	24.85
SPP1	secreted phosphoprotein 1	0.59	26.93	26.19
SSTR2	somatostatin receptor 2	1.09	30.95	31.32
SYK	spleen associated tyrosine kinase	0.86	26.93	26.95
TBP	TATA-box binding protein	0.97	26.97	26.94
TCF20	transcription factor 20	0.89	25.89	25.96
TGFB1	transforming growth factor beta 1	1.09	24.61	24.97
TGFBI	transforming growth factor beta induced	0.96	22.99	22.96
TGFBR2	transforming growth factor beta receptor 2	0.54	25.60	24.95
THBS1	thrombospondin 1	0.61	23.96	23.28
THBS2	thrombospondin 2	0.97	31.96	31.94
THBS3	thrombospondin 3	0.83	29.97	29.73
TIAM1	TIAM Rac1 associated GEF 1	1.09	26.55	26.92
TIMP1	TIMP metallopeptidase inhibitor 1	0.53	25.87	24.99
		0.54	25.60	24.97
	**Mean (RQ)**	**0.54**		
TIMP2	TIMP metallopeptidase inhibitor 2	0.81	24.98	24.69
		0.83	24.99	24.97
	**Mean (RQ)**	**0.82**		
TIMP3	TIMP metallopeptidase inhibitor 3	0.79	22.97	22.64
TIMP4	TIMP metallopeptidase inhibitor 4	0.64	29.96	29.55
TMPRSS4	transmembrane serine protease 4	0.80	25.97	25.90
TNC	tenascin C	1.35	27.50	27.96
TNFSF10	TNF superfamily member 10	0.36	25.99	24.75
TP53	tumor protein p53	0.78	26.98	26.86
TPBG	trophoblast glycoprotein	0.72	24.96	24.73
TSHR	*thyroid stimulating hormone receptor*	*No amplification*	*38.93*	
TWIST1	twist family bHLH transcription factor 1	0.57	29.57	28.99
UBC	ubiquitin C	0.82	20.94	20.69

VCAM1	vascular cell adhesion molecule 1	0.02	38.71	32.97
VCAN	versican	0.76	34.33	33.95
VEGFA	vascular endothelial growth factor A	0.86	24.95	24.97
VEGFC	vascular endothelial growth factor C	0.67	27.97	27.63
VTN	vitronectin	1.18	38.69	38.95
WISP1	cellular communication network factor 4	0.43	38.95	37.98

Target genes with a Ct value ≥ 33 or an expression change of less than 20 % were excluded, with the exception of VEGFA and MMPs. Among the target genes with altered expression, 13 showed increased expression, while 46 showed decreased expression in knockout (KO) cells. * endogenous control.

Supplementary Table S2. Differentially expressed genes in A2058 melanoma cells with PCDHGC3 knockout.

Target gene	Name	RQ	Ct KO	Ct control
* 18S	18S rRNA	1.06	8.99	8.85
		1.15	8.98	8.84
	**Mean (RQ)**	**1.10**		
* ACTB	Actin beta	1.42	17.70	17.99
ADAMTS1	ADAM metallopeptidase with thrombospondin type 1 motif 1	2.05	27.99	28.81
ADAMTS13	ADAM metallopeptidase with thrombospondin type 1 motif 13	1.57	29.53	29.96
ADAMTS8	ADAM metallopeptidase with thrombospondin type 1 motif 8	0.48	40.23	38.96
APC	APC regulator of WNT signaling pathway	1.25	24.94	24.92
B2M	beta-2-microglobulin	1.64	20.45	20.95
BRMS1	BRMS1 transcriptional repressor and anoikis regulator	1.23	25.97	25.94
CASP8	caspase 8	0.88	26.94	26.42
CCL7	*C-C motif chemokine ligand 7*	*No amplification*		*39.97*
CD44	CD44 molecule (Indian blood group)	2.88	20.65	21.95
		2.45	21.97	22.93
	**Mean (RQ)**	**2.66**		
CD82	CD82 molecule	0.65	27.95	26.99
CDH1	cadherin 1	0.01	38.69	31.98
		0.01	36.95	29.96
	**Mean (RQ)**	**0.01**		
CDKN2A	cyclin dependent kinase inhibitor 2A	1.91	21.95	22.55
CEACAM1	CEA cell adhesion molecule 1	0.34	26.86	24.96
CLEC3B	C-type lectin domain family 3 member B	1.60	35.52	35.97
CNTN1	contactin 1	0.15	28.94	25.98
COL11A1	collagen type XI alpha 1 chain	0.37	36.94	35.30
COL12A1	collagen type XII alpha 1 chain	0.74	31.95	31.29
COL14A1	collagen type XIV alpha 1 chain	0.84	27.45	26.97
COL15A1	collagen type XV alpha 1 chain	2.56	23.82	24.96
COL16A1	collagen type XVI alpha 1 chain	0.39	30.96	29.36
COL1A1	collagen type I alpha 1 chain	0.15	35.93	32.98
COL4A2	collagen type IV alpha 2 chain	2.25	21.97	22.92
COL5A1	collagen type V alpha 1 chain	0.01	39.27	32.98
COL6A1	collagen type VI alpha 1 chain	3.22	23.99	25.45
COL6A2	collagen type VI alpha 2 chain	1.67	24.97	25.49
COL7A1	collagen type VII alpha 1 chain	0.97	29.23	28.97
COL8A1	collagen type VIII alpha 1 chain	1130.59	26.97	36.89
CTBP1	C-terminal binding protein 1	1.29	27.96	27.99
CTGF	cellular communication network factor 2	1.94	27.92	28.65
CTNNA1		1.80	21.97	22.60

	catenin alpha 1	1.66	21.95	22.34
	**Mean (RQ)**	**1.73**		
CTNNB1	catenin beta 1	0.75	19.61	18.97
CTNND1	catenin delta 1	1.49	23.61	23.97
CTNND2	*catenin delta 1*	*No amplification*		*27.99*
CTSK	cathepsin K	1.46	21.93	22.15
CXCL12	*C-X-C motif chemokine ligand 12*	*No amplification*		*32.94*
CXCR4	C-X-C motif chemokine receptor 4	0.02	35.90	30.12
DAPK1	death associated protein kinase 1	1.31	25.97	26.03
DCC	*DCC netrin 1 receptor*	*No amplification*		
ECM1	extracellular matrix protein 1	0.45	25.97	24.60
EPCAM	epithelial cell adhesion molecule	0.41	38.59	36.97
EPHB2	EPH receptor B2	0.30	34.96	32.86
ERBB2	erb-b2 receptor tyrosine kinase 2	1.98	25.99	26.64
ETV4	ETS variant transcription factor 4	1.20	24.97	24.90
FAT1	FAT atypical cadherin 1	3.11	24.63	25.93
FGF2	fibroblast growth factor 2	3.82	29.98	31.58
FGFR4	fibroblast growth factor receptor 4	0.38	30.69	28.97
FN1	fibronectin 1	3.32	19.44	20.94
		2.99	19.72	20.97
	**Mean (RQ)**	**3.16**		
FXYD5	FXYD domain containing ion transport regulator 5	1.04	22.95	22.68
* GAPDH	glyceraldehyde-3-phosphate dehydrogenase		17.97	17.75
			18.98	18.64
GNRH1	gonadotropin releasing hormone 1	1.07	29.99	29.76
* GUSB	glucuronidase beta	1.42	22.66	22.94
		1.37	22.84	22.95
	**Mean (RQ)**	**1.39**		
HAS1	hyaluronan synthase 1	2158.54	36.13	46.99
HGF	hepatocyte growth factor	1.24	40.96	40.94
HMBS	hydroxymethylbilane synthase	0.95	25.96	25.67
* HPRT1	hypoxanthine phosphoribosyltransferase 1	1.23	23.87	23.95
		1.22	23.94	23.90
	**Mean (RQ)**	**1.23**		
HPSE	heparanase	0.44	31.97	30.45
HRAS	HRas proto-oncogene. GTPase	1.10	24.98	24.79
HTATIP2	HIV-1 Tat interactive protein 2	0.74	24.72	23.94
ICAM1	intercellular adhesion molecule 1	0.92	25.98	25.64
IGF1	insulin like growth factor 1	0.01	39.91	32.96
IL18	interleukin 18	0.04	37.92	32.80
IL1B	interleukin 1 beta	0.18	32.77	29.95
ITGA1	integrin subunit alpha 1	0.27	25.96	23.84
ITGA2	integrin subunit alpha 2	2.28	28.98	29.95

ITGA3	integrin subunit alpha 3	0.70	26.72	25.97
ITGA4	integrin subunit alpha 4	2.10	26.96	27.81
ITGA5	integrin subunit alpha 5	1.05	25.95	25.80
ITGA6	integrin subunit alpha 6	1.87	23.95	24.62
ITGA7	integrin subunit alpha 7	0.15	26.90	23.96
ITGA8	integrin subunit alpha 8	9.69	32.90	35.95
ITGAL	integrin subunit alpha L	0.03	38.00	32.63
ITGAM	integrin subunit alpha M	2.40	31.93	32.97
ITGAV	integrin subunit alpha V	1.12	23.95	23.90
ITGB1	integrin subunit beta 1	1.46	22.59	22.92
ITGB2	integrin subunit beta 2	0.56	33.96	32.91
ITGB3	integrin subunit beta 3	1.07	23.96	23.84
		1.02	24.26	23.96
	**Mean (RQ)**	**1.05**		
ITGB4	integrin subunit beta 4	1.94	34.99	35.72
ITGB5	integrin subunit beta 5	2.15	22.98	23.86
KAL1	*anosmin 1*	*No amplification*		
KISS1	KiSS-1 metastasis suppressor	0.55	39.99	38.79
KISS1R	KISS1 receptor	3.69	36.97	38.52
KRAS	KRAS proto-oncogene. GTPase	1.11	25.95	25.78
LAMA1	laminin subunit alpha 1	0.44	25.38	23.97
LAMA2	laminin subunit alpha 2	1.28	31.85	31.99
LAMA3	laminin subunit alpha 3	2.12	29.93	30.80
LAMB1	laminin subunit beta 1	2.15	22.03	22.91
		1.95	22.97	23.60
	**Mean (RQ)**	**2.05**		
LAMB3	laminin subunit beta 3	0.36	29.95	28.25
LAMC1	laminin subunit gamma 1	2.33	21.97	22.97
LYPD3	LY6/PLAUR domain containing 3	0.56	37.99	36.82
MCAM	melanoma cell adhesion molecule	1.22	22.98	22.93
MET	MET proto-oncogene, receptor tyrosine kinase	0.25	25.95	23.60
MGAT5	alpha-1.6-mannosylglycoprotein 6-beta-N-acetylglucosaminyltransferase	0.77	25.65	24.94
MMP1	matrix metallopeptidase 1	2.41	20.93	21.97
		2.20	19.95	20.75
	**Mean (RQ)**	**2.30**		
MMP10	matrix metallopeptidase 10	2.29	33.98	34.95
		2.21	32.90	33.71
	**Mean (RQ)**	**2.25**		
MMP11	matrix metallopeptidase 11	1.91	29.97	30.69
MMP12	matrix metallopeptidase 12	4.08	31.97	33.78
MMP13	matrix metallopeptidase 13	0.73	38.61	37.95
MMP14	matrix metallopeptidase 14	1.40	22.70	22.96
		1.33	22.87	22.94
	**Mean (RQ)**	**1.37**		

MMP15	matrix metallopeptidase 15	4.94	24.89	26.97
MMP16	matrix metallopeptidase 16	4.88	25.92	27.98
MMP2	matrix metallopeptidase 2	2.06	22.96	23.78
		2.45	22.96	23.92
	**Mean (RQ)**	**2.25**		
MMP3	matrix metallopeptidase 3	5.68	33.67	35.96
		6.76	33.52	35.94
	**Mean (RQ)**	**6.22**		
MMP7	matrix metallopeptidase 7	0.0002	46.13	33.58
		No amplification		*37.95*
MMP8	matrix metallopeptidase 8	1.54	26.55	26.95
MMP9	matrix metallopeptidase 9	15.47	34.97	38.70
		4.29	34.97	36.73
	**Mean (RQ)**	**9.88**		
MTA1	metastasis associated 1	1.67	24.98	25.39
MTA2	metastasis associated 1 family member 2	1.24	23.98	23.95
MTSS1	MTSS I-BAR domain containing 1	0.36	29.94	28.15
MYC	MYC proto-oncogene. bHLH transcription factor	1.86	23.98	24.55
NCAM1	neural cell adhesion molecule 1	2.41	24.94	25.99
		2.29	26.99	27.85
	**Mean (RQ)**	**2.35**		
NF2	NF2. moesin-ezrin-radixin like (MERLIN) tumor suppressor	1.35	24.82	24.92
NME1	NME/NM23 nucleoside diphosphate kinase 1	1.58	26.60	26.93
NR4A3	nuclear receptor subfamily 4 group A member 3	0.15	27.93	24.88
PECAM1	platelet and endothelial cell adhesion molecule 1	0.14	41.00	37.99
		0.01	43.98	36.28
	**Mean (RQ)**	**0.08**		
PGK1	phosphoglycerate kinase 1	0.74	21.61	20.95
PNN	pinin. desmosome associated protein	1.37	22.83	22.95
PPIA	peptidylprolyl isomerase A	1.74	18.39	18.96
PSCA	prostate stem cell antigen	0.63	34.97	33.96
PTEN	phosphatase and tensin homolog	1.43	25.76	25.95
PTGS2	prostaglandin-endoperoxide synthase 2	0.39	29.65	27.96
RB1	RB transcriptional corepressor 1	2.03	26.25	26.93
RBL1	RB transcriptional corepressor like 1	1.75	24.95	25.42
RBL2	RB transcriptional corepressor like 2	1.06	24.96	24.71
RET	*ret proto-oncogene*	*No amplification*		*38.97*
RHOC	ras homolog family member C	1.10	28.98	28.78
RPLP0	ribosomal protein lateral stalk subunit P0	1.43	19.66	19.96
S100A4	S100 calcium binding protein A4	12.69	26.96	30.29

SELE	*selectin E*	*No amplification*		*39.99*
SELL	selectin L	2.64	28.77	29.95
SELP	*selectin P*	*No amplification*		*38.95*
SERPINB5	serpin family B member 5	0.62	38.96	37.93
SERPINE1	serpin family E member 1	0.33	34.90	32.96
SET	SET nuclear proto-oncogene	1.35	24.84	24.93
SGCE	sarcoglycan epsilon	1.37	23.73	23.97
SMAD2	SMAD family member 2	1.13	24.98	24.82
SMAD4	SMAD family member 4	1.27	24.97	24.98
SNCG	synuclein gamma	0.13	35.97	32.65
SPARC	secreted protein acidic and cysteine rich	2.08	19.14	19.97
SPG7	SPG7 matrix AAA peptidase subunit, paraplegin	0.93	23.96	23.64
SPP1	secreted phosphoprotein 1	0.57	21.93	20.88
SSTR2	somatostatin receptor 2	1.09	29.96	29.75
SYK	*spleen associated tyrosine kinase*	*No amplification*		*31.95*
TBP	TATA-box binding protein	1.66	26.46	26.97
TCF20	transcription factor 20	1.30	25.91	25.96
TGFB1	transforming growth factor beta 1	1.00	24.99	24.65
TGFBI	transforming growth factor beta induced	4.19	28.94	30.78
TGFBR2	transforming growth factor beta receptor 2	2.12	24.19	24.94
THBS1	thrombospondin 1	4.53	24.00	25.96
THBS2	thrombospondin 2	2.13	20.97	21.83
THBS3	thrombospondin 3	1.43	29.68	29.97
TIAM1	TIAM Rac1 associated GEF 1	3.54	23.93	25.42
TIMP1	TIMP metallopeptidase inhibitor 1	1.90	22.27	22.98
		1.62	22.97	23.33
	**Mean (RQ)**	**1.76**		
TIMP2	TIMP metallopeptidase inhibitor 2	1.98	19.97	20.73
		1.64	20.58	20.96
	**Mean (RQ)**	**1.81**		
TIMP3	TIMP metallopeptidase inhibitor 3	0.80	23.52	22.97
TIMP4	TIMP metallopeptidase inhibitor 4	1.98	36.95	37.61
TMPRSS4	*transmembrane serine protease 4*	*No amplification*		
TNC	tenascin C	4.74	25.97	28.00
TNFSF10	TNF superfamily member 10	0.08	38.90	34.97
TP53	tumor protein p53	1.87	24.40	24.97
TPBG	trophoblast glycoprotein	0.02	33.66	27.95
TSHR	*thyroid stimulating hormone receptor*	*No amplification*		*39.98*
TWIST1	twist family bHLH transcription factor 1	1.81	26.98	27.51
UBC	ubiquitin C	1.27	21.83	21.96
VCAM1	*vascular cell adhesion molecule 1*	*No amplification*	*35.95*	
VCAN	versican	9.45	26.97	29.98

VEGFA	vascular endothelial growth factor A	1.52	26.96	27.23
VEGFC	vascular endothelial growth factor C	1.79	27.97	28.48
VTN	vitronectin	0.62	32.89	31.99
WISP1	cellular communication network factor 4	0.05	35.98	31.18

Target genes with a Ct value ≥ 33 or an expression change of less than 20 % were excluded, with the exception of MMPs. Among the target genes with altered expression, 75 showed increased expression, while 24 showed decreased expression in knockout (KO) cells. * endogenous control.
